# rDNA Copy Number Variation and Methylation During Normal and Premature Aging

**DOI:** 10.1111/acel.14497

**Published:** 2025-01-24

**Authors:** Alva B. C. Geisen, Natalia Santana Acevedo, Junko Oshima, Marcus Dittrich, Ramya Potabattula, Thomas Haaf

**Affiliations:** ^1^ Institute of Human Genetics, Julius Maximilians University Würzburg Germany; ^2^ Department of Pathology University of Washington Seattle Washington USA; ^3^ Department of Bioinformatics Julius Maximilians University Würzburg Germany

**Keywords:** aging, droplet digital PCR, rDNA copy number variation, rDNA promoter methylation, rDNA transcription unit, Werner syndrome

## Abstract

Ribosomal RNA is the main component of the ribosome, which is essential for protein synthesis. The diploid human genome contains several hundred copies of the rDNA transcription unit (TU). Droplet digital PCR and deep bisulfite sequencing were used to determine the absolute copy number (CN) and the methylation status of individual rDNA TU in blood samples of healthy individuals. The absolute CN ranged from 243 to 895 (median 469). There was no difference in absolute CN between males and females and no gain or loss of copies with age (15–71 years). The number of rDNA TU with a completely unmethylated (0%) or lowly methylated (1%–10%) promoter region significantly decreased, whereas the number of copies with higher (11%–100%) methylation increased with age. The number of presumably active TU with a hypomethylated (0%–10%) promoter varied from 94 to 277 (median 180), independent from absolute CN. In contrast, the number of inactive hypermethylated (11%–100%) copies strongly increased with absolute CN. Promoter hypermethylation compensates to some extent for the enormous CN variation among individuals. Patients with Werner syndrome, a premature aging syndrome displayed the same CN variation and age‐related methylation changes as controls. The role of rDNA CN variation as a modulating factor in human health and disease is largely unexplored. In particular, very low and high CN may be associated with increased disease risk.

## Graphical abstract

In > 160 healthy individuals the absolute rDNA copy number (CN) in blood ranged from approximately 240 to 900. The number of active copies with a completely unmethylated promoter varied from 40 to 120, independent of absolute CN. Promoter methylation is a compensation mechanism for absolute CN variation. Aging was associated with a loss of unmethylated active rDNA copies. The role of variation in rDNA dosage and ribosome biogenesis in aging and age‐related disease remains to be elucidated.

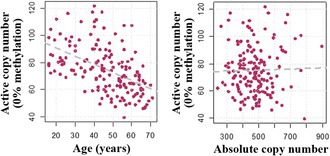

**Abbreviations**
CN: copy number
CP: core promoter
DBS: deep bisulfite sequencing
ddPCR: droplet digital PCR
ETS: external transcribed spacer
rDNA/RNA: ribosomal DNA/RNA
TU: transcription unit
UCE: upstream control element

## 1 Introduction

Human ribosomal DNA (rDNA) is represented in the genome by several hundred rDNA transcription units (TU), which are tandemly arrayed in the nucleolus organizer regions of the acrocentric short arms, allowing mass production of rRNA. The rDNA copy number (CN) can vary by about one power of 10 among individuals, however only part of them may be active (Porokhovnik and Lyapunova 2019; Smirnov et al. 2021). Every TU consists of two parts: a 13 kb long 47S DNA encoding the 18S, 5.8S, and 28S RNAs of ribosomal particles, and a 30 kb long intergenic spacer. The rRNAs along with 70–80 ribosomal proteins form the two subunits of the ribosome, the cellular machinery for messenger RNA translation and protein synthesis (Khatter et al. 2015; Zentner et al. 2011). Ribosome biogenesis is closely interrelated with cell metabolism, growth, proliferation, and the maintenance of homeostasis. Considering the importance of ribosome biogenesis for essentially every cellular process, little is known about the functional consequences of rDNA CN variation, which so far has been largely neglected in genomic medicine. Several studies found that the CN is decreasing with age (Gaubatz, Prashad, and Cutler 1976; Zafiropoulos et al. 2005), whereas in other studies the CN remained unchanged (D'Aquila et al. 2017; Malinovskaya et al. 2018).

Each rDNA TU contains > 1500 CpG sites whose methylation is involved in epigenetic rDNA silencing (Santoro and Grummt 2001; Wang and Lemos 2019). Increased rDNA methylation with age has been observed in different rodent and human somatic tissues (D'Aquila et al. 2017; Flunkert et al. 2018; Oakes et al. 2003; Yang et al. 2024) as well as in germ cells (Potabattula et al. 2020, 2022; Yang et al. 2024). Thus, an epigenetic rDNA methylation clock appears to be conserved in the mammalian soma and germ line (Potabattula et al. 2020; Wang and Lemos 2019). rDNA methylation reflects changes in rDNA transcription, ribosome biogenesis, and nucleolar biology during development, aging, and in many age‐related conditions, including cancer, metabolic, and cardiovascular disorders (Shao et al. 2021; Wang et al. 2015; Yang et al. 2024). In this light, rDNA methylation is a primary candidate when looking for age‐related epigenetic signatures which may have a functional impact on the aging process.

The many contradictory studies on rDNA CN may at least partially be due to the limitations of different techniques used. For absolute CN counting, most studies relied on quantitative PCR, which requires standard curves in each experiment. Alternatively, nonradioactive quantitative hybridization or assembly of rDNA repeats from whole genome data have been used. However, only a fraction, maybe 100 to 200 of all rDNA repeats appear to be active and account for biological functions; the majority of copies in most individuals is silenced by DNA methylation and several histone modifications (Srivastava, Srivastava, and Ahn 2016; Zentner et al. 2011). rDNA methylation is usually determined by bisulfite (pyro)sequencing, which measures average methylation of millions of rDNA TU. Here we have used a combination of droplet digital PCR (ddPCR) for absolute CN counting and deep bisulfite sequencing (DBS) for measuring the methylation status of individual TU. This allows us to determine the number of copies with 0%, 10%, 20%, etc. methylation in a given sample with unprecedented accuracy.

## 2 Results

### 2.1 Global rDNA Methylation Is Driven by Age and Copy Number

**FIGURE 1:**
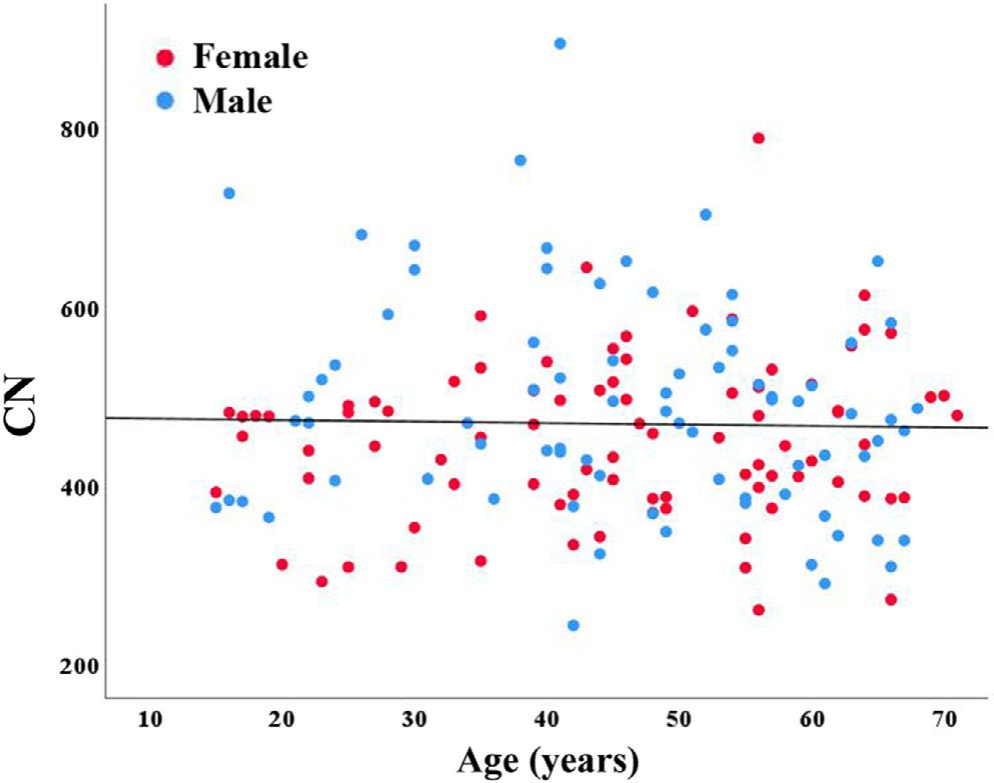
Absolute copy number of rDNA TU does not change with age. Blue dots represent absolute CN in 82 male and red dots in 85 female healthy individuals, ranging from 15 to 71 years in age. There is no significant correlation (*ρ* = −0.02; *p* = 0.80) between CN and age.

Using ddPCR (targeting the 47S rDNA), we determined absolute rDNA CN in blood DNA of 82 male and 85 female healthy individuals. The age ranged from 15 to 71 years (mean ± SD 45.7 ± 14.9) and CN from 243 to 895 (median 469, IQR 130; mean 469 ± 107). There was no significant (Mann–Whitney *U* test; *p* = 0.09) CN difference between males (median 473, IQR 169; mean 487 ± 120) and females (median 453, IQR 116; mean 451 ± 90) (Figure S1A). In addition, there was no significant correlation (Spearman *ρ* = −0.02; *p* = 0.80) between CN and age (Figure 1).

Using DBS, we analyzed average methylation of 25 contiguous CpG sites in a 239 bp region, encompasing the core promoter (CP) and the upstream control element (UCE) as well as of 38 CpGs in a 271 bp region in the 5′ external transcribed spacer (ETS). Neither the CP/UCE (Figure S1B) nor the ETS (Figure S1C) showed a significant (Mann–Whitney *U* test; *p* = 0.55 for CP/UCE and *p* = 0.19 for the ETS) methylation difference between males (median 27, IQR 15; mean 26 ± 10 for the CP/UCE and median 10, IQR 4; mean 11 ± 3 for the ETS) and females (median 26, IQR 12; mean 26 ± 10 for the CP/UCE and median 10, IQR 3; mean 10 ± 3 for the ETS). On average CP/UCE methylation (median 26%; mean 25.9% ± 9.4%) was higher than that of the ETS (median 10%; mean 10.5% ± 2.9%), but methylation of both regions was highly correlated (*ρ* = 0.85; *p* < 0.001) with each other. Moreover, there was a highly significant positive correlation between methylation and age for both the CP/UCE (*ρ* = 0.21; *p* = 0.006) and the ETS region (*ρ* = 0.24; *p* = 0.002) (Figure S2). In addition, DNA methylation of both regions showed a strong positive correlation (*ρ* = 0.80; *p* < 0.001 for CP/UCE and *ρ* = 0.66; *p* < 0.001 for the ETS) with absolute rDNA CN (Figure S3).

### 2.2 Loss of Hypomethylated (0%–10%) rDNA TU Copies With Age

Mean rDNA methylation increased with age and CN. To study these methylation changes in more detail, we classified the CN in different methylation bins (0%, 1%–10%, 11%–20%, 21%–30%, 31%–40%, 41%–50%, 51%–75%, and 76%–100%). Overall, approximately 80–110 rDNA TU per genome were completely unmethylated and 100–200 copies displayed methylation values between 1% and 10% (Figure S4). The remaining TU showed > 10% methylation.

When plotting the number of TU within every bin against age, the number of completely unmethylated TU (CP/UCE *ρ* = −0.57; *p* < 0.001 and ETS *ρ* = −0.31; *p* < 0.001) and lowly (1%–10%) methylated TU (CP/UCE *ρ* = −0.48; *p* < 0.001 and ETS *ρ* = −0.14; *p* = 0.07) was significantly decreasing with age for both the CP/UCE (Figure S5A) and the ETS (Figure S5B). The number of TU with 11%–20% (CP/UCE *ρ* = −0.09; *p* = 0.24 and ETS *ρ* = 0.08; *p* = 0.33) methylation remained more or less unchanged, whereas the number of TU with 21%–30%, 31%–40%, 41%–50%, 51%–75%, and 76%–100% methylation was slightly increasing with age. Although these positive correlations between allele methylation and age were not significant (see legend of Figure S5), it was evident in all bins from 20% to 100% for both the CP/UCE and the ETS region.

**FIGURE 2:**
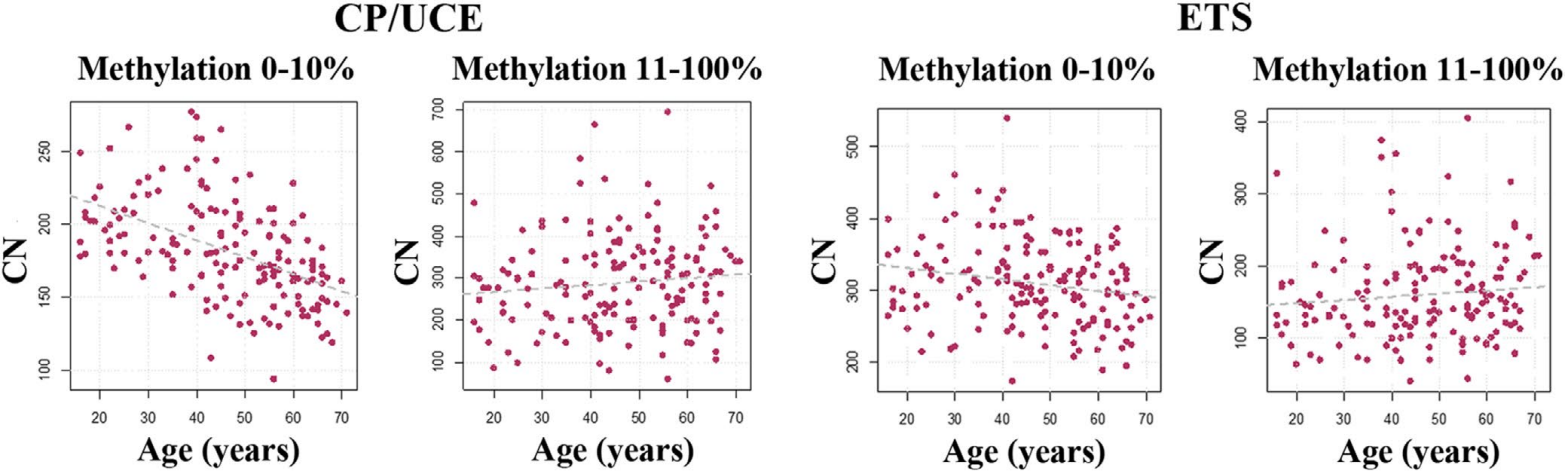
Number of hypomethylated (0%–10%) and hypermethylated (> 10%) rDNA TU is correlated with age. For both the CP/UCE and the ETS region, the number of hypomethylated (0%–10%) copies is significantly (*ρ* = −0.55; *p* < 0.0001 for the CP/UCE and *ρ* = −0.21; *p* = 0.007 for the ETS) decreasing with age, whereas the number of hypermethylated (11%–100%) copies is increasing (*ρ* = 0.11; *p* = 0.15 for CP/UCE and *ρ* = 0.13; *p* = 0.11 for the ETS). Each dot represents an individual control sample (CP/UCE *N* = 166; ETS *N* = 165).

The promoter methylation level plays a crucial role in switching a gene from active to inactive (Santoro and Grummt 2001; Weber et al. 2007). Because it is usually the density of methylated CpGs in a cis‐regulatory region rather than individual CpGs that turns a gene “on” or “off”, TU with one or two methylated CpGs in the CP/UCE region (25 CpGs) and 1–3 methylated CpGs in the ETS region (38 CpGs) were considered as single CpG methylation errors without functional consequences. For the sake of simplicity, we classified TU copies with 0%–10% mean methylation as active and copies with > 10% methylation as hypermethylated and prone to epigenetic silencing. For both the CP/UCE and the ETS region (Figure 2), the number of hypomethylated (0%–10%) active TU was significantly (CP/UCE *ρ* = −0. 55, *p* < 0.0001 and ETS *ρ* = −0.21; *p* = 0.007) decreasing with age. The number of hypermethylated (11%–100%), presumably inactive TU was increasing, however this age effect was not significant (CP/UCE *ρ* = 0.11; *p* = 0.15 and ETS *ρ* = 0.13; *p* = 0.11). As average methylation (of all rDNA TU copies) was considerably higher in the CP/UCE region (median 26%; mean 25.9% ± 9.4%), compared to the ETS (median 10%; 10.5% ± 2.9%), it is not surprising that there were more hypomethylated ETS copies (mean 309 ± 5 8) than CP/UCE copies (182 ± 35). We propose that the number of rDNA TU with a hypomethylated promoter (median 180, range 94–277) represents the number of active TU.

### 2.3 The Number of Hypermethylated (> 10%) rDNA TU Copies Increases With Absolute CN

Next we analyzed the effect of absolute CN on rDNA TU methylation in different methylation bins. Interestingly, the number of rDNA TU with a completely unmethylated (*ρ* = −0.03; *p* = 0.67) or lowly (1%–10%) methylated (*ρ* = 0.09; *p* = 0.25) promoter was not influenced by absolute CN (Figure S6A). In contrast, the number of copies with higher methylation values showed highly significant positive correlations with absolute CN in all bins with > 10% methylation: 11%–20% (*ρ* = 0.50; *p* < 0.0001), 21%–30% (*ρ* = 0.71; *p* < 0.0001), 31%–40% (*ρ* = 0.80; *p* < 0.0001), 41%–50% (*ρ* = 0.84; *p* < 0.0001), 51%–75% (*ρ* = 0.89; *p* < 0.0001), and 76%–100% (*ρ* = 0.86; *p* < 0.0001). For the ETS (Figure S6B), there was a strong positive correlation with absolute CN in all analyzed methylation bins: 0% (*ρ* = 0.70; *p* < 0.0001), 1%–10% (*ρ* = 0.91; *p* < 0.0001), 11%–20% (*ρ* = 0.87; *p* < 0.0001), 21%–30% (*ρ* = 0.79; *p* < 0.0001), 31%–40% (*ρ* = 0.77; *p* < 0.0001), 41%–50% (*ρ* = 0.78; *p* < 0.0001), 51%–75% (*ρ* = 0.79; *p* < 0.0001), and 76%–100% (*ρ* = 0.80; *p* < 0.0001).

**FIGURE 3:**
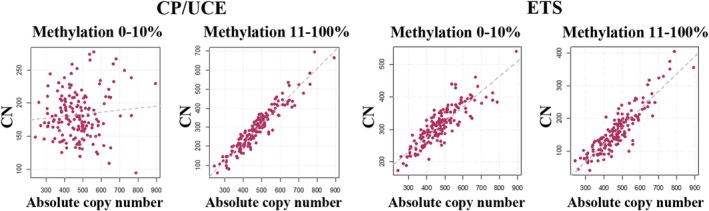
The number of hypermethylated (> 10%) rDNA TU depends on absolute copy number. For the CP/UCE region, the number of hypomethylated copies is slightly (*ρ* = 0.02; *p* = 0.76), if at all increasing with absolute CN, whereas the number of hypermethylated copies is strongly (*ρ* = 0.95; *p* < 0.0001) increasing with absolute CN. For the ETS region, both the number of hypo‐ (*ρ* = 0.87; *p* < 0.0001) and hypermethylated (*ρ* = 0.87; *p* < 0.0001) copies show a strong positive correlation with absolute CN. Each dot represents an individual control sample (CP/UCE *N* = 162 and ETS *N* = 161).

In summary, the number of rDNA TU with a hypomethylated (0%–10%) promoter region was not or little dependent on absolute CN (*ρ* = 0.02; *p* = 0.76), whereas the number of hypermethylated (11%–100%) promoters highly significantly (*ρ* = 0.95; *p* < 0.0001) increased with absolute CN (Figure 3). For the ETS, both hypomethylated (*ρ* = 0.87; *p* < 0.0001) and hypermethylated (*ρ* =0.87; *p* < 0.0001) rDNA TU showed a strong positive correlation with absolute CN. In this context, it is interesting to note, that 9 of the 10 samples with the highest absolute CN were males and only one was female (Figure S7). Of the 10 samples with the lowest CN, four were males and 6 were females. As expected, the high CN group showed a significantly (Mann–Whitney *U* test; *p* < 0.001) higher mean rDNA methylation and significantly (*p* < 0.001) higher number of hypermethylated (11%–100%) copies. There was no significant between‐group difference in the number of hypomethylated (0%–10%) copies and age (see legend of Figure S7).

### 2.4 rDNA CN and Werner Syndrome (WS)

In addition to aging in healthy individuals, we studied rDNA CN and methylation in 24 patients with WS, suffering from pathological aging of several organ systems. The age ranged from 9 to 59 years and absolute CN from 352 to 935 (median 493, IQR 147), which is comparable to healthy controls (median 469, IQR 130). Similar to controls, absolute CN did not significantly (*ρ* = 0.38; *p* = 0.06) change with age (Figure S8A). Promoter methylation appeared to increase with age (Figure S8B); however, due to the small sample size this age effect was not significant (*ρ* = 0.18; *p* = 0.41). In contrast, promoter methylation was significantly (*ρ* = 0.77; *p* < 0.001) correlated with absolute CN (Figure S8C). There were no CN or methylation differences between *WRN*, *LMNA*, and *POLD1*‐mutation carriers. Mean methylation of the CP/UCE region in WS was 26.5% ± 12.5%, almost identical to that in controls (25.9% ± 9.4%).

**FIGURE 4:**
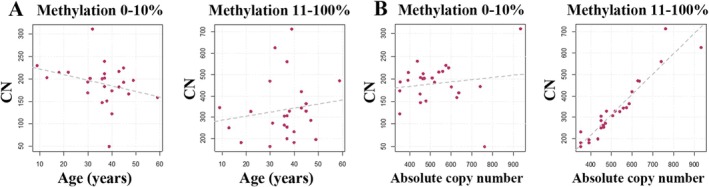
rDNA TU methylation and CN in Werner syndrome. (A) Diagrams showing that the number of hypomethylated (0%–10%) alleles is decreasing (*ρ* = −0.34; *p* = 0.06) and the number of hypermethylated (> 10%) CP/UCE regions is increasing (*ρ* = 0.15; *p* = 0.47) with age, respectively. (B) The number of hypomethylated promoters is slightly, if at all increasing (*ρ* = 0.08; *p* = 0.70) with absolute CN, whereas the number of hypermethylated copies is strongly (*ρ* = 0.97; *p* < 0.0001) increasing. Each dot represents an individual WS patient (*N* = 24).

The number of completely unmethylated (0%) or lowly (1%–10%) methylated CP/UCE regions decreased with age, whereas the CN with 11%–20%, 21%–30%, 31%–40%, 41%–50%, 51%–75%, and 76%–100% promoter methylation increased (Figure S9A). Again because of small sample size, these results were not significant. Similar to controls, the number of hypomethylated (0%–10%) promoter regions was negatively (*ρ* = −0.38; *p* = 0.06) correlated with age, whereas the number of hypermethylated (> 10%) CP/UCE regions showed a positive (*ρ* = 0.15; *p* = 0.46) correlation with age (Figure 4A).

There was no apparent effect of absolute CN on the number of unmethylated (*ρ* = −0.08; *p* = 0.69) and lowly methylated (*ρ* = 0.17; *p* = 0.43) promoters in WS (Figure S9B). In contrast, the number of alleles with > 10% methylation was significantly increasing with absolute CN: 11%–20% (*ρ* = 0.53; *p* = 0.007), 21%–30% (*ρ* = 0.71; *p* = 0.0001), 31%–40% (*ρ* = 0.73; *p* < 0.0001), 41%–50% (*ρ* = 0.76; *p* < 0.0001), 51%–75% (*ρ* = 0.85; *p* < 0.0001), and 76%–100% (*ρ* = 0.76; *p* < 0.0001). Overall, the number of hypomethylated (0%–10%) promoters remained stable (*ρ* = 0.08; *p* = 0.70) and the number of hypermethylated (11%–100%) promoters significantly (*ρ* = 0.97; *p* < 0.0001) increased with absolute CN (Figure 4B). Interestingly, two of 24 WS samples exhibited extreme absolute (764 and 936) and active (49 and 311) CN.

## 3 Discussion

Most published data show a positive correlation between global rDNA methylation and aging across different tissues and species (D'Aquila et al. 2017; Flunkert et al. 2018; Oakes et al. 2003; Potabattula et al. 2020, 2022; Wang and Lemos 2019; Yang et al. 2024). However, in this context it is important to note that global rDNA methylation is a surrogate marker, representing millions of individual rDNA TU. A global DNA methylation of 10% can be due to 1 of 10 CpGs being methylated in all copies or all CpGs being methylated in 10% of copies. Moreover, previous studies did not take into account the enormous CN variation among individuals.

### 3.1 Absolute rDNA CN Remain Stable, Hypomethylated CN Decrease, and Hypermethylated CN Increase During Aging

Our results show that global rDNA methylation depends on age, absolute CN, and the region studied. The absolute number of rDNA TU appears to remain stable from adolescence to old age. Using a combination of ddPCR and DBS, we determined the number of rDNA TU copies within different methylation ranges. For both the promoter (CP/UCE) and the ETS, the number of unmethylated (0%) and lowly (1%–10%) methylated TU significantly decreased with age. The number of hypermethylated copies with 21%–30%, 31%–40%, 41%–50%, 51%–75%, and 76%–100% methylation increased with age, although the results were not significant. For the promoter region, which is the most important regulatory element for rDNA transcription, the number of hypomethylated copies did not appear to depend on absolute CN. In contrast, the number of hypermethylated copies was strongly increasing with absolute CN. For the ETS, both hypo‐ and hypermethylated rDNA TU showed a strong positive correlation with absolute CN. In this light, ETS methylation may not be an absolute requirement for rDNA silencing, but represent a secondary phenomenon. Spreading of promoter methylation into the neighboring 5′ ETS may help to stabilize the regional methylation patterns.

In our control cohort, absolute CN ranged from 243 to 895 (median 469; mean 469 ± 107). The number of active rDNA TU with a hypomethylated promoter region was much less variable, ranging from 94 to 277 (median 180; mean 182 ± 35). On average across all samples, about 40% of all rDNA TU were hypomethylated and presumably active. However, the percentage of active CN strongly depends on absolute CN. In individuals with low absolute CN (< 300) about 60% of rDNA TU were active, whereas in individuals with high absolute CN (> 600) < 30% were active. Evidently, promoter hypermethylation is a compensation mechanism for the enormous CN variation among individuals.

### 3.2 rDNA CN in Werner Syndrome

WS (Oshima, Sidorova, and Monnat Jr. 2017) is not only characterized by genome instability but also appears to be a transcription disease. The WRN helicase is involved in transcription enhancement by RNA polymerase II (RNAPII). Transcription efficiency is reduced to 40%–60% in WS cell lines (Balajee et al. 1999). Previously, we identified several hundred genes which are differentially methylated in WS and enriched with molecular functions linked to transcription factor activity and sequence‐specific DNA binding to promoters transcribed by RNAPII (Maierhofer et al. 2019). Here we show that both absolute CN and the number of hypomethylated presumably active rDNA copies are comparable between WS (absolute CN median 493, IQR 451–598) and healthy controls (median 469, IQR 390–520). Similar to controls, the number of hypomethylated copies was decreasing and the number of hypermethylated copies was increasing with age in WS. Two of 24 WS patients in our cohort displayed extreme absolute and active rDNA CN, however this may be a chance/coincidence and not be a general feature of WS.

### 3.3 CN Variation and Disease

Most individuals are endowed with 300–600 rDNA TU (median 469, IQR 390–520), although individuals with 200 or > 1000 copies have been observed. The absolute CN range is maintained by a unique recombination and CN control system (Hori, Engel, and Kobayashi 2023). The higher the absolute CN the more copies are inactivated by promoter hypermethylation. Despite the essential role of rDNA activity and ribosomes for cell function, relatively little is known about the effects of extreme CN variation on human diseases. There may be a minimum number of active CN required for protein synthesis in embryonal and fetal development (Morton et al. 2023). In this light, studies on rDNA CN in embryos, which arrest growth during preimplantation development, and spontaneous abortions are urgently needed.

Malinovskaya et al. (2018) reported that the range of CN variation was narrower in individuals older than 72 years. One plausible explanation is that individuals with very low or very high CN do not reach old age. In our cohort, ranging from 15 to 71 years in age, very low and very high CN were equally distributed in all age groups. Accumulating evidence suggests that high CN (in blood samples) is associated with an increased schizophrenia risk and severity (Chestkov et al. 2018; Ershova et al. 2022; Li et al. 2023). One study (Pietrzak et al. 2011) reported that rDNA silencing occurs in the brain during early stages of Alzheimer disease pathogenesis, while the absolute CN remains unchanged. Compared to healthy tissue, many cancers display consistent rDNA methylation changes (Shao et al. 2021). Hypomethylation of the rDNA promoter is thought to facilitate proliferation of cancer cells (Ghoshal et al. 2004; Zhou et al. 2016). Moreover, CN instability has been observed in some cancers (Valori et al. 2020; Wang and Lemos 2017).

It has been proposed that CN and rDNA activity can modulate multifactorial disease risk (Porokhovnik and Lyapunova 2019). Most age‐related diseases, including metabolic, cardiovascular, and neurodevelopmental disorders as well as cancers have a multifactorial origin, involving complex patterns of inheritance. Recently, absolute CN number has been correlated with body mass index in adult humans (Law et al. 2024). Interestingly, absolute CN was not associated with rRNA transcription rates in adult tissues. This is consistent with our observation that the number of active rDNA TU with a hypomethylated promoter is largely independent of absolute CN. Because rDNA CN and methylation are difficult to quantify by currently used molecular diagnostic techniques, the importance of rDNA CN in health and disease has been largely neglected so far.

### 3.4 Limitations

Although the age range in our study is relatively wide (15–71 years), it does not include individuals at very young or very old age. Most models assume a linear relationship between age and rDNA methylation. In our data set individuals with extreme absolute CN are found in all age groups. However, we cannot exclude that variation of absolute CN is reduced in octogenarians or nonagenarians, as proposed in the literature (Malinovskaya et al. 2018) or whether an active CN in the medium range is advantageous for healthy aging or not. Similarly, we do not know what happens during development from the intrauterine period to puberty.

Blood cell composition is a potential confounder in methylation analysis because it changes with advancing age and different cell types may exhibit different methylation trajectories (Marttila et al. 2024). Numerous studies (D'Aquila et al. 2017; Flunkert et al. 2018; Oakes et al. 2003; Potabattula et al. 2020, 2022; Wang and Lemos 2019; Yang et al. 2024) have shown that global rDNA methylation is increasing with age in different somatic tissues/cell types and germ cells of humans, rodents and other mammalian species. This argues in favor of the notion that the low but significant correlation between global rDNA methylation and age observed in this study reflects an evolutionarily conserved interrelation between rDNA methylation and age, rather than changes in blood cell composition. For both, the CP/UCE and the ETS the number of hypomethylated (0%–10%) rDNA TU was significantly decreasing with age, whereas the number of hypermethylated (> 10%) copies was increasing. This strongly suggests that aging is associated with a loss of hypomethylated active rDNA copies. However, as we do not have blood cell counts or methylation array data (which allow one to correct for differing blood cell composition), we cannot exclude the formal possibility that the observed age‐related methylation changes are at least to some extent influenced by changes in blood cell composition.

The main aim of our study was to analyze rDNA methylation changes with age at the single allele level and determine the number of alleles within defined methylation bins. We show that the number of copies with a hypomethylated (0%–10%) promoter region is not affected by absolute CN. Consistent with a CN compensation mechanism, the number of hypermethylated copies strongly increases with absolute CN. Since we do not have RNA samples or cell lines of our study subjects we cannot study the functional consequences, that is, whether these hypomethylated copies are really transcribed. Moreover, it is difficult to prove by functional experiments, which methylated CpG density is required for epigenetic silencing and there may not be a sharp threshold. However, there is convincing evidence from the literature (Santoro and Grummt 2001; Potapova et al. 2024) that the activity of rRNA genes is epigenetically regulated by promoter methylation. This does not exclude that other mechanisms such as chromatin structure also play a role in rDNA silencing.

## 4 Conclusions

ddPCR and DBS allow one to accurately determine the absolute CN of rDNA TU as well as the number of copies within a given methylation range. In blood samples of 82 male and 85 female healthy individuals absolute CN ranged from 243 to 895 (median 469, IQR 390–520). Absolute CN did not depend on age (15–71 years) or sex, however promoter methylation was increasing with age. The number of active copies with a hypomethylated (0%–10%) promoter region ranged from 94 to 277 (median 180; mean 182 ± 35) and was decreasing with age. The number of copies with hypomethylated promoter did not appear to be affected by absolute CN, whereas the number of hypermethylated rDNA TU strongly increased with absolute CN. In individuals with low absolute CN about 60%, whereas in individuals with high absolute CN < 30% of rDNA TU were hypomethylated and presumably active. Both absolute CN and hypomethylated CN show enormous variation, which must make sense in the light of evolution (Dobzhansky 1973). Variation in rDNA dosage and ribosome biogenesis may provide an additional layer of genetic (absolute CN) and epigenetic (active CN) variability which can enhance phenotypic plasticity and adaptations to different environments. So far the role of rDNA CN variation in complex phenotypes and age‐related disease is largely underestimated.

## 5 Materials and Methods

### 5.1 Study Samples

The study on human blood DNA samples was approved by the Ethics Committee at the Medical Faculty of Würzburg University (no. 20220718 01). Informed consent was obtained from all individuals participating in this study. The DNA samples from peripheral blood were anonymized excess materials from genetic diagnostics and isolated using the classical salting out method. The 82 males and 85 females without mutation in predictive diagnostics were considered as healthy controls. The age range was from 15 to 71 years. The blood DNAs of 18 classical WS patients with *WRN* mutations, three atypical WS patients each with *LMNA* and *POLD1* mutations, respectively, were obtained through the International Registry of Werner Syndrome (http://www.wernersyndrome.org). The age range was from 9 to 59 years. For more details see Table S4 in Maierhofer et al. (2019).

The DNA concentration and purity were determined using a NanoDrop 2000c spectrophotometer (ThermoScientific, Massachusetts, USA). All the DNA samples underwent bisulfite conversion using the EpiTect Fast 96 Bisulfite kit (Qiagen, Hilden, Germany), and the converted DNA was preserved at −20°C for subsequent use.

### 5.2 Droplet Digital PCR

ddPCR primers (Table S1) for the 28S rDNA and the TATA‐box binding protein (*TBP*) gene, which served as reference gene (internal control), were taken from the literature (Xu et al. 2017). To evaluate the CN of 28S rDNA, 1 ng of genomic DNA (per sample) was added to a mixture containing 10 μL of ddPCR Supermix for Probes (without dUTP, 2× concentrated), 1 μL each of 20× FAM (reference gene label) and 20× HEX (rDNA label) assay mixes, 0.2 μL of Hae III restriction endonuclease (10,000 units/mL; New England Biolabs, Frankfurt, Germany), and 6.8 μL of H_2_O. Probes labeled with distinct fluorophores for rDNA and reference gene, respectively, enabled precise quantification of both loci with a single duplex ddPCR reaction. Furthermore, restriction enzyme digestion of genomic DNA facilitated the separation of tandem copies of rDNA, largely reducing sample viscosity and enhancing template accessibility for downstream analyses (Salim and Gerton 2019). Droplet generation using the QX200 droplet generator was performed according to the manufacturer's protocol (Bio‐Rad, Feldkirchen, Germany), followed by an endpoint PCR. Amplifications were carried out with an initial denaturation at 95°C for 10 min, 40 cycles of 96°C for 30 s, 40 cycles of 54°C for 56 s, and a final step at 98°C for 10 min. Subsequently, a QX200 droplet reader (Bio‐Rad) was used to detect the signal from individual droplets and the data analyses were executed using QX Manager 1.2 Standard Edition software (Bio‐Rad).

### 5.3 Deep Bisulfite Sequencing

DBS primers (Table S2) were designed for two assays targeting different loci within the rDNA TU, each containing an A/G variant (Babaian 2017; Potabattula et al. 2020). Assay 1 includes 25 contiguous CpGs covering the CP and UCE; assay 2 encompasses 38 CpGs and targets the 5′ ETS. For the CP/UCE variant (GRCh37; chr13: 999.905), the majority (> 60%) of samples displayed only the major G allele; in about 40% of samples on average 9% of the reads represented the minor G allele. For the ETS variant (chr13: 1.000.139), about 25% of the samples showed only the major A allele; 75% of samples were endowed with both variants and on average 23% of reads representing the minor G allele.

First‐round PCRs were carried out in 50 μL reactions consisting of 5 μL 10× PCR buffer with MgCl_2_, 1 μL (10 mM) of PCR grade nucleotide mixture, 2.5 μL (10 pmol/mL) of forward and reverse primer each, 0.4 μL (5 U/μL) FastStart Taq DNA polymerase, 2 μL (~50 ng) bisulfite‐converted DNA, and 36.6 μL ddH_2_O. Completely unmethylated (0%) and completely methylated (100%) DNAs (Qiagen) served as controls for assessing the reliability of methylation measurements for each DBS amplicon. PCR products were cleaned with Agencourt AMPure XP beads (Beckmann Coulter, Krefeld, Germany), quantified using the Qubit dsDNA BR Assay system kit (Invitrogen, Karlsruhe, Germany), and diluted to a concentration of 0.2 ng/μL. In the second PCRs, the two different amplicons for each sample were pooled together and barcoded with multiple identifiers (MIDs), using NEBNext Multiplex Oligos for Illumina (Dual Index Primers Set 1 and 2). Touchdown PCR thermocycler conditions were adapted to provide homogenous amplification of PCR templates of varying sizes. The purified and quantified PCR pools were diluted to a concentration of 4 nM, and 3 μL of this dilution from each of the MIDs were pooled together into one final pool for next‐generation sequencing (NGS).

NGS was performed using the MiSeq platform (Illumina, California, USA) and Reagent Kit V2 (500 cycles) cartridge (Illumina) according to the manufacturer's instructions. Sequencing was carried out with 250 bp paired‐end sequencing. After the run, the sequencing reads were processed by Illumina Genome Analyzer. FASTQ files were analyzed further using the Amplikyzer2 software (Leitao et al. 2018), which provides a detailed nucleotide‐level analysis including the calculation of methylation rates (per amplicon) at both single nucleotide and regional levels. Initially, all sequences were aligned to the reference genomic sequence of each amplicon using default settings. For the subsequent extraction of reads and CpG‐wise methylation status, only reads with an overall bisulfite conversion rate of > 95% were considered and further downstream processing of Amplikyzer output files was performed. To avoid an effect of genetic variation within the rDNA TU on our methylation results, only reads representing the major variant, carrying a G allele for the CP/UCE and an A allele for the ETS, were used for methylation analyses.

### 5.4 Statistical Analysis

Both descriptive and inferential statistical analyses were conducted using IBM SPSS software version 28 and R (version 3.6.3). For correlation analyses among variables such as absolute CN, mean methylation, and age, Spearman's correlations were applied. For group comparisons, based on the data distribution, nonparametric Mann–Whitney *U* tests were performed. The number of rDNA copies within a given methylation range (i.e., active copies with 0%–10% methylation) was determined by multiplying the absolute CN (from ddPCR) with the percentage of reads (from DBS) in the corresponding methylation bin (i.e., 0%–10%). A *p* < 0.05 was considered as statistically significant throughout the analyses.

## Author Contributions

T.H. designed the study. A.B.C.G. and N.S.A. performed the experiments. R.P. supervised the experimental work. R.P. and M.D. did bioinformatic analyses. J.O. provided WS samples. All authors were involved in data collection, interpretation and revision of the manuscript.

## Conflicts of Interest

The authors declare no conflicts of interest.

## Supporting information


**Figure S1.** rDNA TU copy number and methylation do not depend on sex.
**Figure S2.** Mean methylation of the rDNA TU increases with age.
**Figure S3.** Mean methylation of the rDNA TU increases with absolute copy number.
**Figure S4.** Number and methylation distribution of reads.
**Figure S5.** Age‐related methylation changes of the rDNA CP/UCE and the ETS.
**Figure S6.** Interrelation of rDNA CP/UCE and ETS methylation and absolute copy number.
**Figure S7.** Extreme rDNA copy numbers.
**Figure S8.** rDNA promoter methylation in Werner syndrome depends on age and absolute CN.
**Figure S9.** Effects of age and absolute CN on rDNA promoter methylation in Werner syndrome.
**Table S1.** Primers for ddPCR of the human ribosomal DNA region.
**Table S2.** Primers for deep bisulfite sequencing of the human rDNA TU.

## Data Availability

All data are contained in the manuscript and its Supporting Information.
