# Supplementary material for: rDNA Copy Number Variation and Methylation During Normal and Premature Aging
Source: Aging Cell. 2025 Jan 24;24(5):e14497. doi: 10.1111/acel.14497 (PMC12073889; doi:10.1111/acel.14497)
Supplement: Supplementary file 1 — Figure S1. rDNA TU copy number and methylation do not depend on sex. Figure S2. Mean methylation of the rDNA TU increases with age. Figure S3. Mean methylation of the rDNA TU increases with absolute copy number. Figure S4. Number and methylation distribution of reads. Figure S5. Age‐related methylation changes of the rDNA CP/UCE and the ETS. Figure S6. Interrelation of rDNA CP/UCE and ETS methylation and absolute copy number. Figure S7. Extreme rDNA copy numbers. Figure S8. rDNA promoter methylation in Werner syndrome depends on age and absolute CN. Figure S9. Effects of age and absolute CN on rDNA promoter methylation in Werner syndrome. Table S1. Primers for ddPCR of the human ribosomal DNA region. Table S2. Primers for deep bisulfite sequencing of the human rDNA TU. [file ACEL-24-e14497-s001.docx]

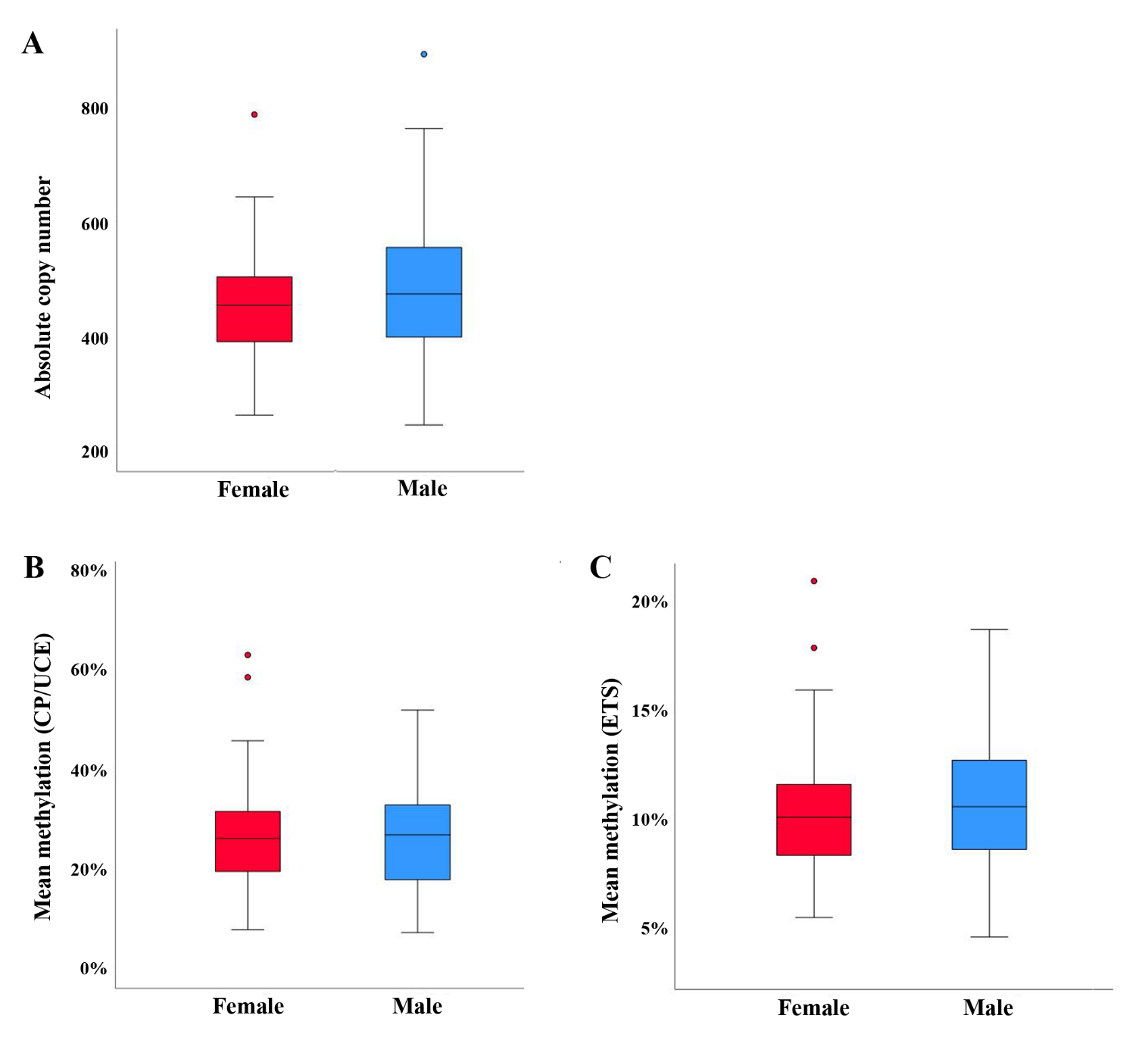


**Figure S1.** rDNA TU copy number and methylation do not depend on sex. Comparisons of absolute CN (A) and methylation of the CP/UCE (B) and ETS (C), respectively, between 85 females (red) and 82 males (blue). The median is presented by a horizontal line. The bottom of the box indicates the 25^th^ and the top the 75^th^ percentile. Outliers are indicated by circles. Please note that methylation of the CP/UCE region is higher than that of the ETS region.


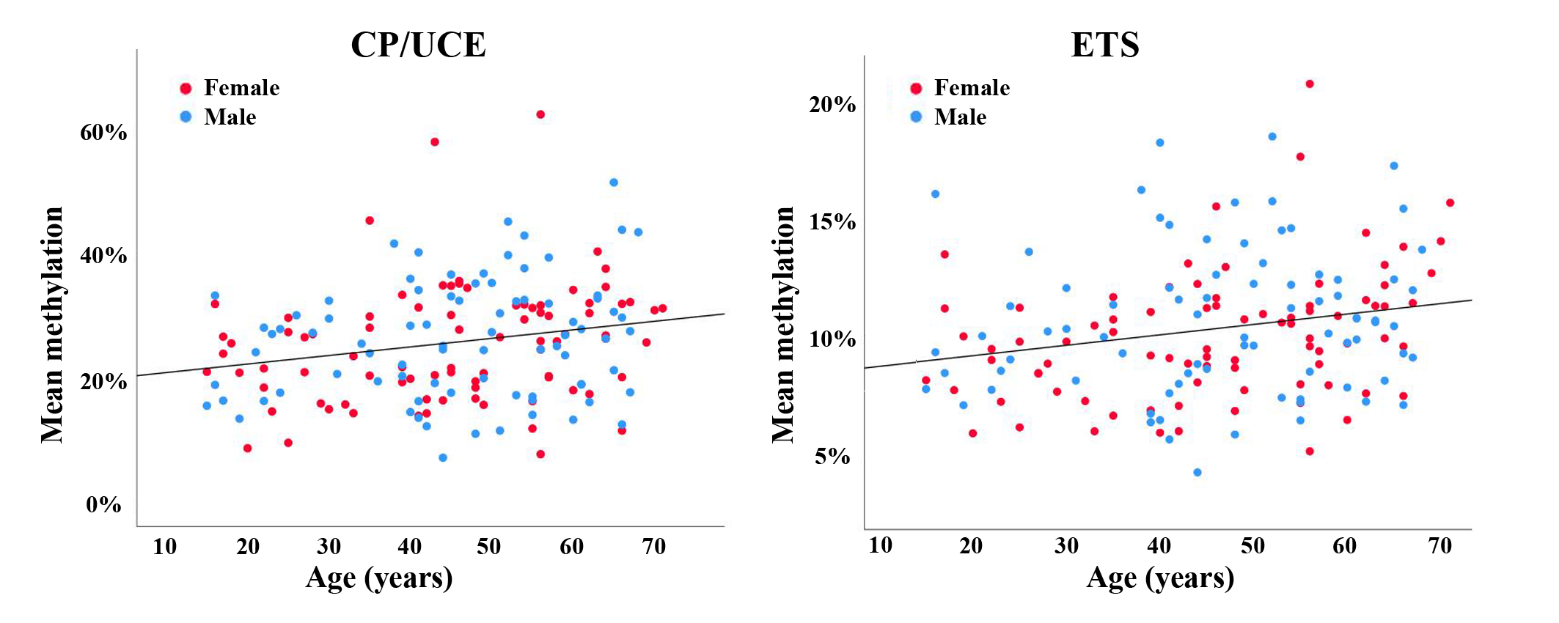


**Figure S2.** Mean methylation of the rDNA TU increases with age. Blue dots represent mean methylation (Y axis) of the CP/UCE and the ETS region, respectively, in 82 males and red dots in 85 females, ranging from 15 to 71 years in age (X axis). Both CP/UCE (*ρ* = 0.21; *p* = 0.006) and ETS (*ρ* = 0.24; *p* = 0.002) methylation are positively correlated with age.


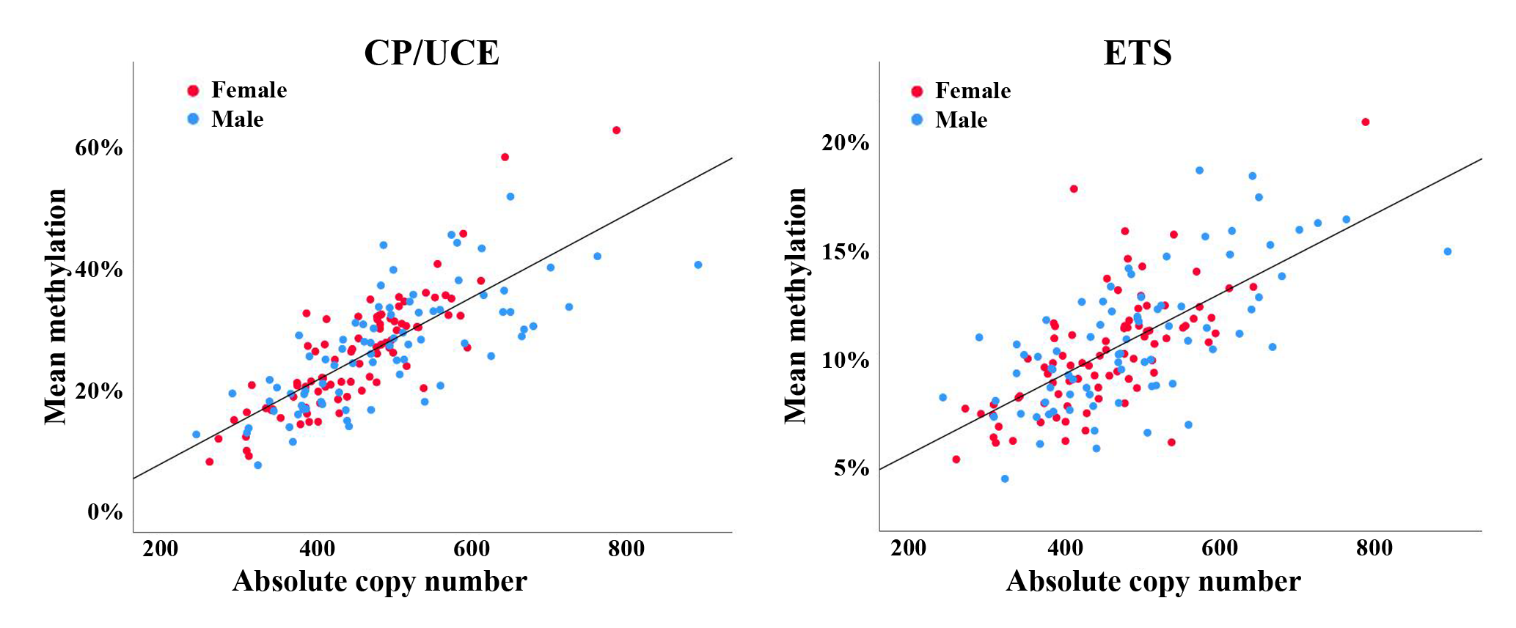


**Figure S3.** Mean methylation of the rDNA TU increases with absolute copy number. Blue dots represent mean methylation (Y axis) of the CP/UCE and the ETS region, respectively, in 82 males and red dots in 85 females with absolute CN (on the X axis) ranging from 234 to 895. There is a strong positive correlation between methylation and absolute CN for both the CP/UCE (*ρ* = 0.80; *p* < 0.001) and the ETS (*ρ* = 0.66; *p* < 0.001).


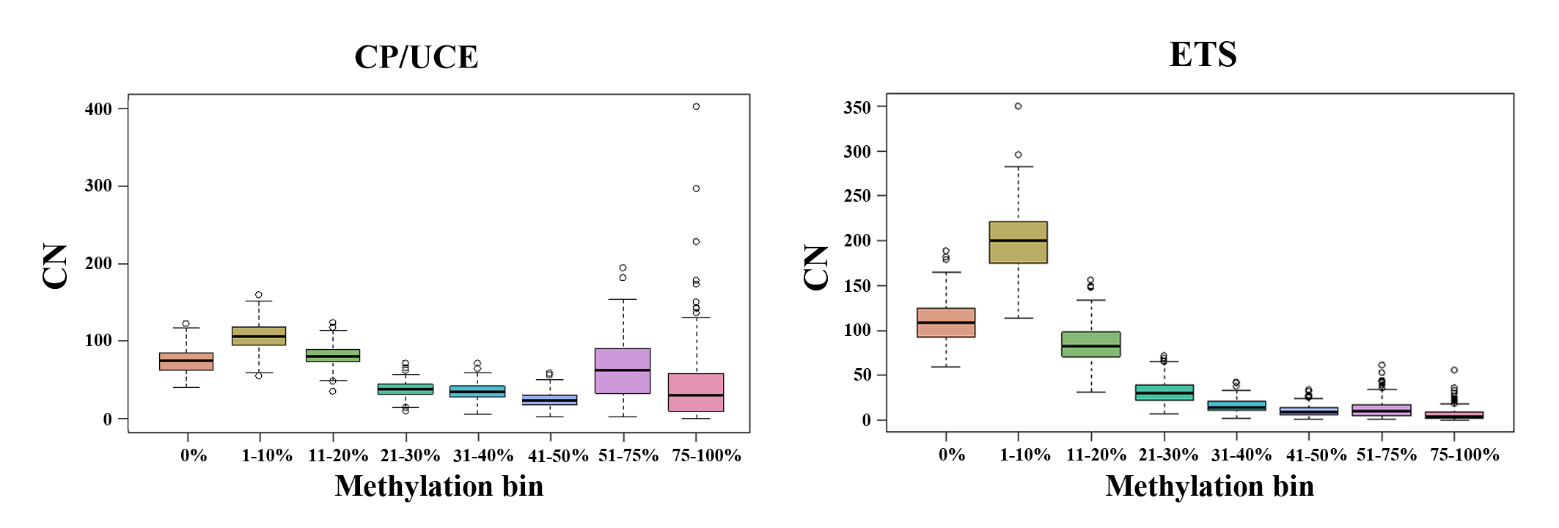


**Figure S4.** Number and methylation distribution of reads. Box plots showing the CN (on the Y axis) within a given methylation range in the CP/UCE (N = 166) and ETS (N = 165) in control samples. Methylation bins representing 0%; 1-10%, 11-20%, 21-30%, 31-40%, 41-50%, 51-75%, and 76-100% are indicated on the X axis. The median is presented by a horizontal line. The bottom of the box indicates the 25^th^ and the top the 75^th^ percentile. Outliers are indicated by open circles.


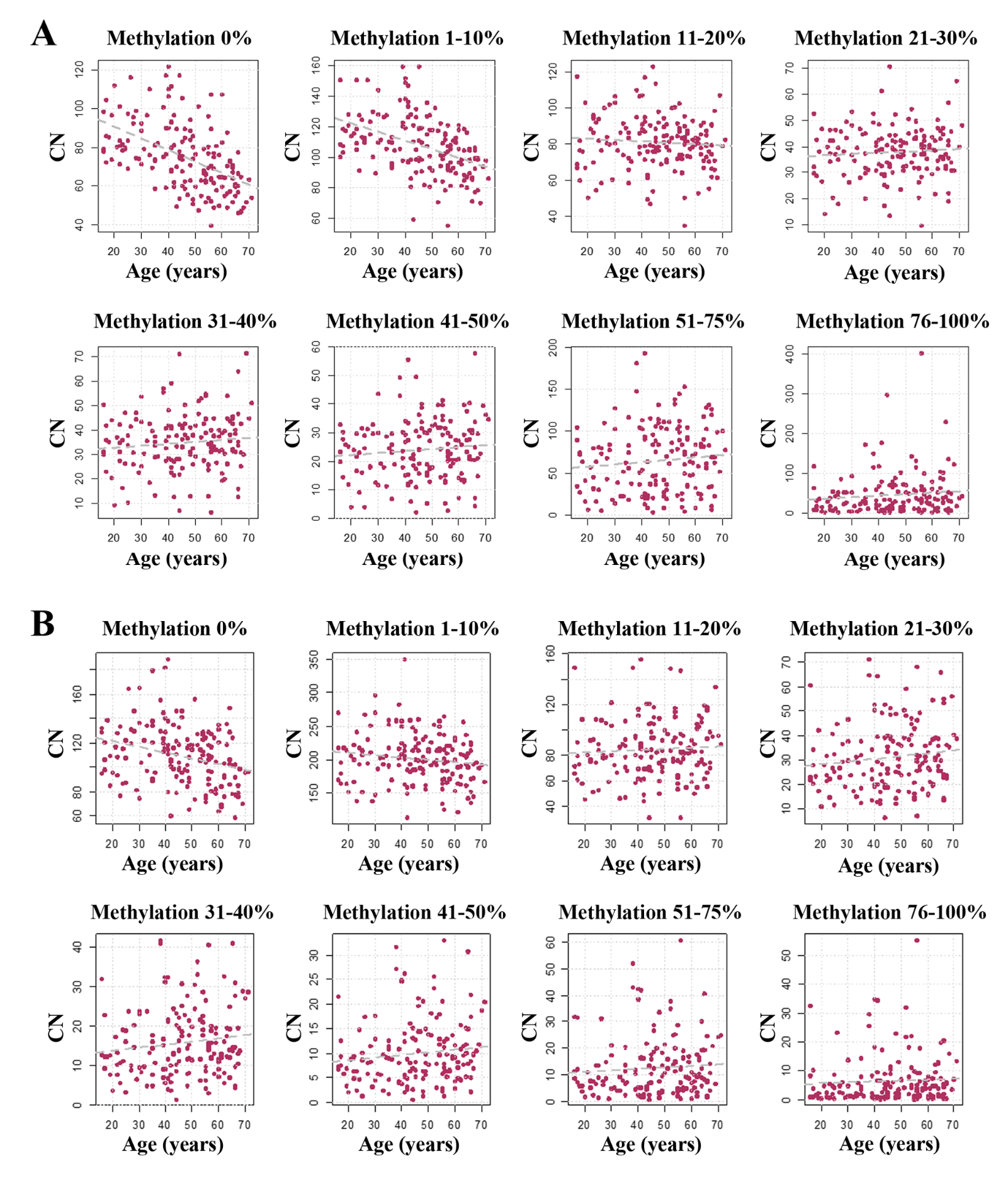


**Figure S5.** Age-related methylation changes of the rDNA CP/UCE (A) and the ETS (B). The Y axis shows the number of rDNA TU within a given methylation bin. The first bin represents completely unmethylated TU. The following bins represent mean methylation (across the entire CP/UCE and ETS region, respectively) from 1-10%, 11-20%, 21-30%, 31-40%, 41-50%, 51-75%, and 76-100%. Each dot represents an individual sample (CP/UCE N = 166; ETS N = 165). Please note that for both the CP/UCE and the ETS the number of TU with low (0-10%) promoter methylation decreases with age, whereas the number of TU with high methylation (11-100%) is increasing. For the CP/UCE (A), the Spearman correlations in the analyzed bins are as follows: 0% (*ρ* = -057; *p* < 0.0001), 1-10% (*ρ* = -0.48; *p* < 0.0001), 11-20% (*ρ* = -0.09; *p* = 0.24), 21-30% (*ρ* = 0.06; *p* = 0.48), 31-40% (*ρ* = 0.09; *p* = 0.26), 41-50% (*ρ* = 0.10; *p* = 0.20), 51-75% (*ρ* = 0.10; *p* = 0.20), and 76-100% (*ρ* = 0.12; *p* = 0.13). For the ETS (B), the Spearman correlations are the following: 0% (*ρ* = -0.31; *p* < 0.0001), 1-10% (*ρ* = -0.14; *p* = 0.07), 11-20% (*ρ* = 0.08; *p* = 0.33), 21-30% (*ρ* = 0.14; *p* = 0.09), 31-40% (*ρ* = 0.13 *p* = 0.10), 41-50% (*ρ* = 0.14; *p* = 0.08), 51-75% (*ρ* = 0.11; *p* = 0.16), and 76-100% (*ρ* = 0.11; *p* = 0.15).


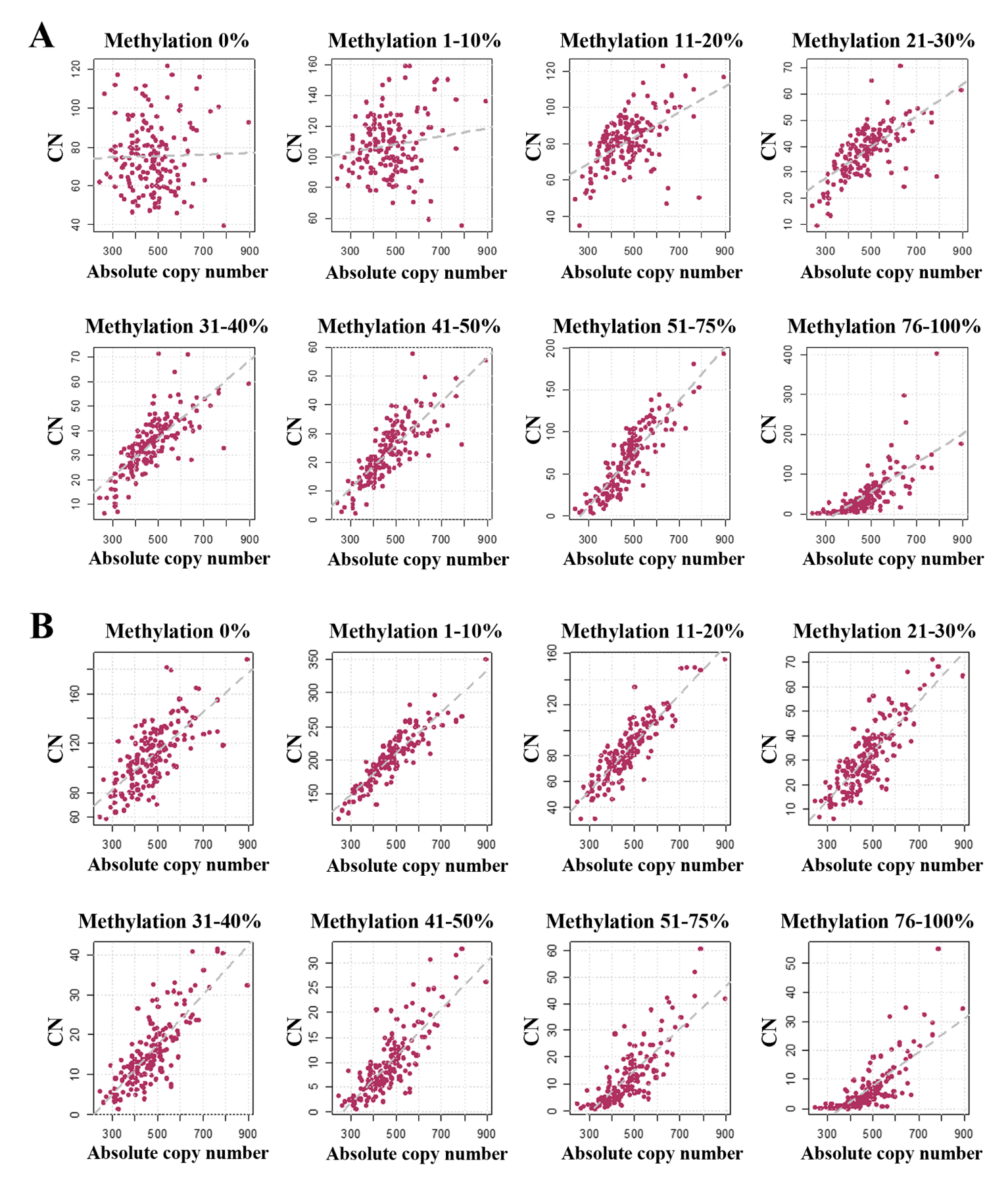


**Figure S6.** Interrelation of rDNA CP/UCE (A) and ETS (B) methylation and absolute copy number. Each dot represents an individual sample (CP/UCE N = 162 and ETS N = 161). The X axis shows the absolute number of rDNA TU copies. The Y axis presents the number of rDNA TU within a given methylation bin (0%, 1-10%, 11-20%, 21-30%, 31-40%, 41-50%, 51-75%, and 76-100%). For the promoter region (A), the number of completely unmethylated and lowly (1-10%) methylated copies is comparable across all samples, independent of absolute CN. In all other methylation bins, the number of copies within a given methylation range increases with absolute CN. The Spearman correlations for the CP/UCE (A) are as follows: 0% (*ρ* = -0.03; *p* = 0.25), 1-10% (*ρ* = 0.09; *p* = 0.67), 11-20% (*ρ* = 0.50; *p* < 0.0001), 21-30% (*ρ* = 0.71; *p* < 0.0001), 31-40% (*ρ* = 0.80; *p* < 0.0001), 41-50% (*ρ* = 0.84; *p* < 0.0001), 51-75% (*ρ* = 0.89; *p* < 0.0001), and 76-100% (*ρ* = 0.86; *p* < 0.0001). For the ETS (B), the number of copies in each methylation bin (0%, 1-10%, 11-20%, 21-30%, 31-40%, 41-50%, 51-75%, and 76-100%) increases with absolute CN. The Spearman correlations are the following: 0% (*ρ* = 0.70; *p* < 0.0001), 1-10% (*ρ* = 0.91; *p* < 0.0001), 11-20% (*ρ* = 0.87; *p* < 0.0001), 21-30% (*ρ* = 0.79; *p* < 0.0001), 31-40% (*ρ* = 0.77; *p* < 0.0001), 41-50% (*ρ* = 0.78; *p* < 0.0001), 51-75% (*ρ* = 0.79; *p* < 0.0001), and 76-100% (*ρ* = 0.80; *p* < 0.0001).


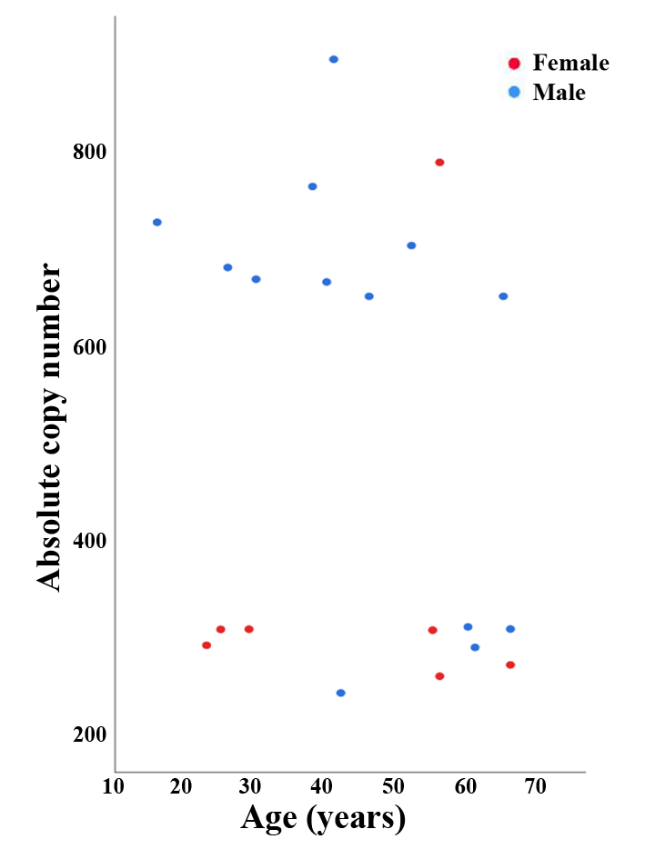


**Figure S7.** Extreme rDNA copy numbers. Abundance (9 of 10) of males (blue dots) in samples with very high (> 600) absolute CN. In contrast, 6 of 10 samples with very low (< 300) CN are females (red dots). There is no significant difference in age between samples with high vs. low CN.

|  | **Low CN group** | | **High CN group** | | **Between group-difference*** |
| --- | --- | --- | --- | --- | --- |
|  | **Mean±SD** | **Range** | **Mean± SD** | **Range** | ***p* value** |
| **Age (years)** | 48.3±17.1 | 23-66 | 41±14.6 | 16-56 | 0.28 |
| **Sex ratio (male vs. female) (%)** | 40 |  | 90 |  | 0.06 |
| **Mean methylation (%)** | 13±3.1 | 8-19 | 39.2±11 | 29-63 | < 0.001 |
| **Number of hypomethylated copies** | 174±21.5 | 146-219 | 203.3±56.5 | 95-267 | 0.12 |
| **Number of hyper-methylated copies** | 115.5±27.2 | 59-146 | 516±102.3 | 406-694 | < 0.001 |

*Mann-Whitney U test


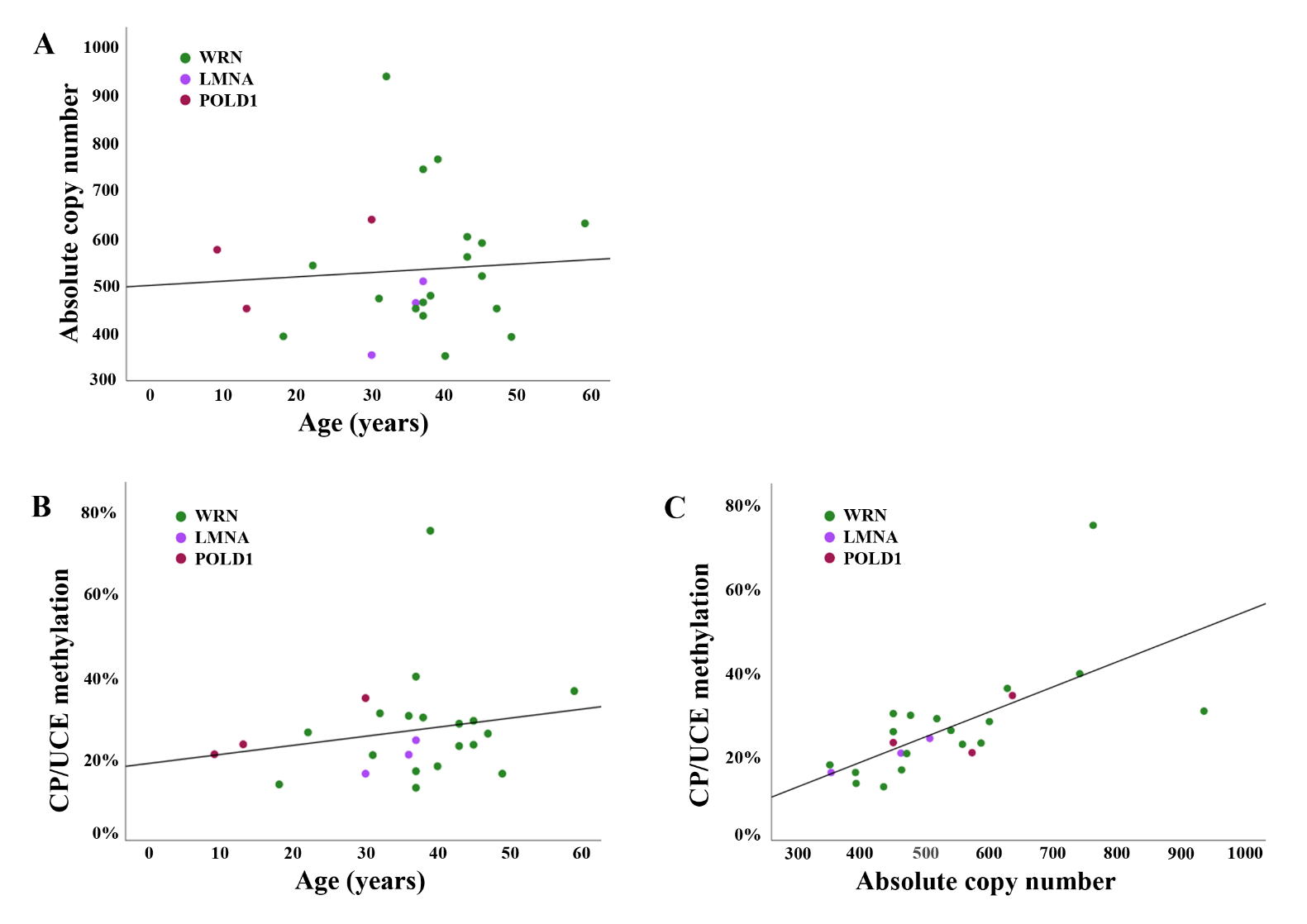


**Figure S8.** rDNA promoter methylation in Werner syndrome depends on age and absolute CN. (A) Absolute copy number (*ρ* = 0.38; *p* = 0.06) and (B) promoter methylation (*ρ* = 0.18; *p* = 0.41) are not significantly correlated with age in WS patients. (C) Promoter methylation is strongly (*ρ* = 0.77; *p* < 0.001) correlated with absolute CN. Patients with *WRN*, *LMNA*, and *POLD1* mutations are indicated by green, mauve, and red dots.


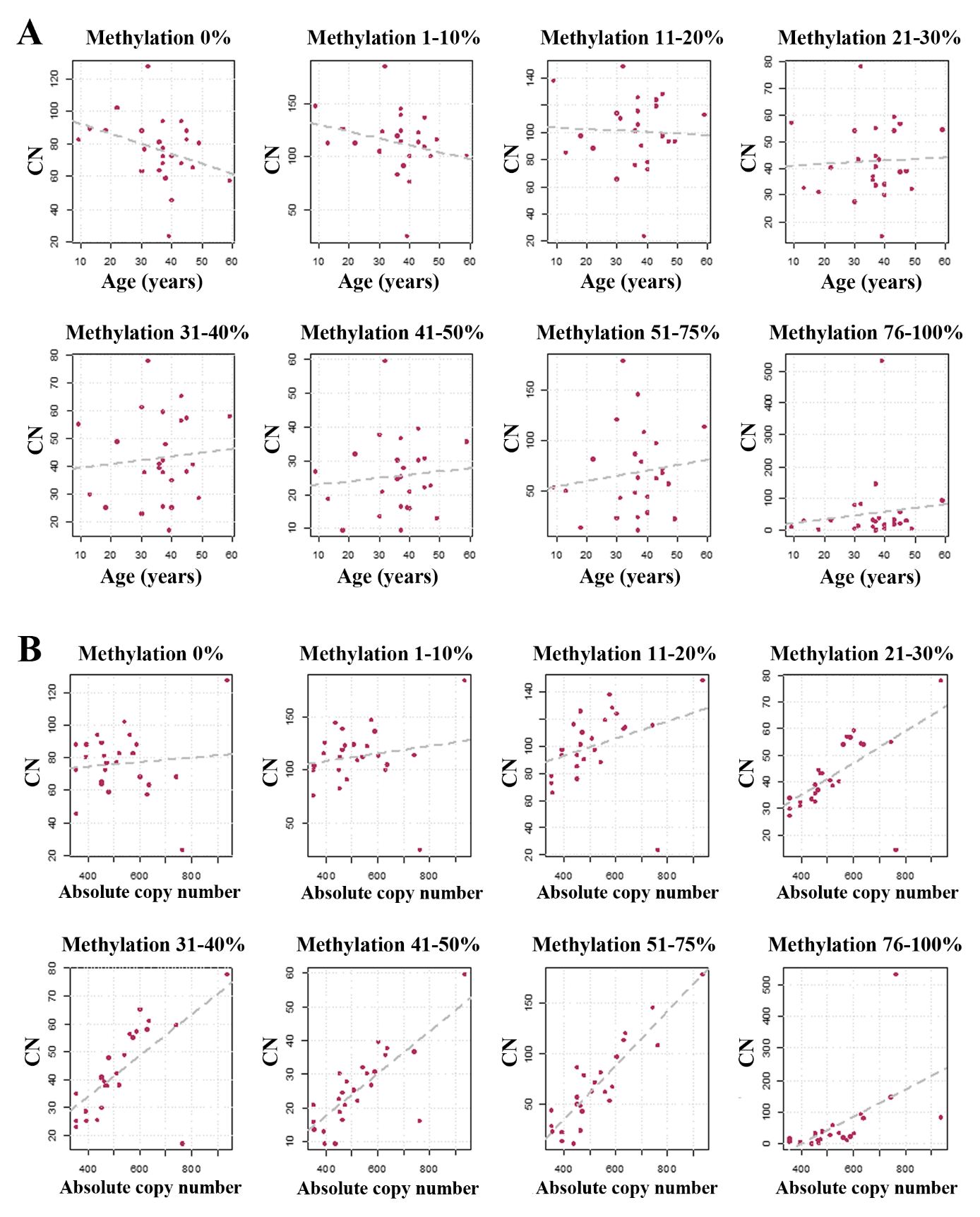


**Figure S9.** Effects of age (A) and absolute CN (B) on rDNA promoter methylation in Werner syndrome. (A) The upper diagram shows age-related methylation changes of the rDNA CP/UCE in 24 WS patients (red dots). The Y axis shows the number of rDNA TU within a given methylation bin. The number of TU with a completely unmethylated promoter (*ρ* = -0.36 *p* = 0.08) or a lowly methylated (0-10%) promoter (*ρ* = -0.29; *p* = -0.16) decreases with age, whereas the number of TU with higher methylation is increasing: 11-20% (*ρ* = 0.01; *p* = 0.96), 21-30% (*ρ* = 0.08; *p* = 0.71), 31-40% (*ρ* = 0.09; *p* = 0.67), 41-50% (*ρ* = 0.06; *p* = 0.79), 51-75% (*ρ* = 0.11; *p* = 0.60), and 76-100% (*ρ* = 0.13; *p* = 0.52). (B) The lower diagram presents the interrelation of rDNA CP/UCE methylation and absolute CN in WS. The X axis shows the absolute number of rDNA TU copies. The Y axis presents the number of rDNA TU within a given methylation bin. The number of completely unmethylated (*ρ* = -0.08; *p* = 0.68) and lowly methylated (*ρ* = 0.17; *p* = 0.43) copies remains relatively stable, whereas the number of copies with > 10% methylation is significantly increasing with absolute copy number: 11-20% (*ρ* = 0.53; *p* = 0.007), 21-30% (*ρ* = 0.71; *p* = 0.0001), 31-40% (*ρ* = 0.73; *p* < 0.0001), 41-50% (*ρ* = 0.76; *p* < 0.0001), 51-75% (*ρ* = 0.85; *p* < 0.0001), and 76-100% (*ρ* = 0.76; *p* < 0.0001).

**Table S1.** Primers for ddPCR of the human ribosomal DNA region.

| **Assay** | **Primer** | **Sequence (5'-3')** |
| --- | --- | --- |
| 28S rDNA | Forward | AACGTGAGCTGGGTTTAG |
|  | Reverse | CTCGTACTGAGCAGGATTAC |
|  | Probe | 5HEX/TGGCAACAA/ZEN/CACATCATCAGT/3IABkFQ |
| *TBP* | Forward | GATATGAGACTGTGGGTAAGT |
|  | Reverse | GATCCTTTGAACACCCTAATG |
|  | Probe | 56-FAM/ACAGAGATC/ZEN/ACTGCAGTTGC/3IABkFQ |

**Table S2.** Primers for deep bisulfite sequencing of the human rDNA TU.

| **Assay** | **Primer** | **Sequence (5'-3')** | **Amplicon length** | **No. of**  **CpGs** | **Annealing Temp. (^o^C)** |
| --- | --- | --- | --- | --- | --- |
| rDNA  CP and UCE | Forward | TATTYGGAGGTTTAATTTTTTTAG | 239 bp | 25 | 56^o^C |
|  | Reverse | TATATCCTAAAATTAACCAAAAAACCCC |  |  |  |
| rDNA ETS | Forward | GGAGTTAGYGGGGTGGGGTTGT | 271 bp | 38 | 56^o^C |
|  | Reverse | ACTAAAAAAATTAAACCTCC |  |  |  |
